# High dose craniospinal irradiation as independent risk factor of permanent alopecia in childhood medulloblastoma survivors: cohort study and literature review

**DOI:** 10.1007/s11060-022-04186-2

**Published:** 2022-11-12

**Authors:** C. Satragno, A. Verrico, F. Giannelli, A. Ferrero, S. Campora, M. Turazzi, F. Cavagnetto, I. Schiavetti, M. L. Garrè, F. Garibotto, C. Milanaccio, G. Piccolo, M. Crocco, A. Ramaglia, S. Di Profio, S. Barra, L. Belgioia

**Affiliations:** 1 Dipartimento Di Medicina Sperimentale (DIMES), Università Degli Studi Di Genova, Via Leon Battista Alberti, 16132 Genova, GE Italia; 2 Unità di Neuroncologia, IRCCS Istituto Giannina Gaslini, Genova, Italia; 3 Dipartimento di Scienza Della Salute (DISSAL), Università Degli Studi di Genova, Genova, Italia; 4 UO Radioterapia Oncologica, IRCCS Ospedale Policlinico San Martino, Genova, Italia; 5 UO Fisica Sanitaria, IRCCS Ospedale Policlinico San Martino, Genova, Italia; 6 Dipartimento di Scienze Della Salute (DISSAL), Sezione di Biostatistica, Università Degli Studi di Genova, Genova, Italia; 7 Dipartimento di Neuroscienze, Riabilitazione, Oftalmologia, Genetica, Ginecologia e Pediatria (DINOGMI), Università Degli Studi di Genova, Genova, Italia; 8 Unità di Neuroradiologia, IRCCS Istituto Giannina Gaslini, Genova, Italia; 9 Unità di Psicologia, IRCCS Istituto Giannina Gaslini, Genova, Italia

**Keywords:** Permanent alopecia, Medulloblastoma survivors, Craniospinal irradiation, Scalp dose, Hair follicle dose-sensibility

## Abstract

**Purpose:**

Our aim was to determine the main risk factors related to the occurrence of permanent alopecia in childhood medulloblastoma (MB) survivors.

**Methods:**

We retrospectively analyzed the clinical features of all consecutive MB survivors treated at our institute. We divided the patients into 3 groups depending on the craniospinal irradiation (CSI) dose received and defined permanent alopecia first in terms of the skin region affected (whole scalp and nape region), then on the basis of the toxicity degree (G). Any relationship between permanent alopecia and other characteristics was investigated by a univariate and multivariate analysis and Odds ratio (OR) with confidence interval (CI) was reported.

**Results:**

We included 41 patients with a mean10-year follow-up. High dose CSI resulted as an independent factor leading to permanent hair loss in both groups: alopecia of the whole scalp (G1 p-value 0.030, G2 p-value 0.003) and of the nape region (G1 p-value 0.038, G2 p-value 0.006). The posterior cranial fossa (PCF) boost volume and dose were not significant factors at multivariate analysis neither in permanent hair loss of the whole scalp nor only in the nuchal region.

**Conclusion:**

In pediatric patients with MB, the development of permanent alopecia seems to depend only on the CSI dose ≥ 36 Gy. Acute damage to the hair follicle is dose dependent, but in terms of late side effects, constant and homogeneous daily irradiation of a large volume may have a stronger effect than a higher but focal dose of radiotherapy.

**Supplementary Information:**

The online version contains supplementary material available at 10.1007/s11060-022-04186-2.

## Introduction

Medulloblastoma (MB) is the most common Central Nervous System (CNS) embryonal tumor in children [1], accounting for approximately 20% of all pediatric CNS tumors. It typically arises in the posterior cranial fossa and metastasizes along the neuroaxis [2].

Although MB is a malignant brain tumor, survival rates at 5-years from diagnosis can reach up to 80% for the standard-risk patients and close to 100% for WNT-activated biomolecular subgroup [2, 3].

Standard treatment involves surgery and adjuvant therapy, which includes radiotherapy (RT) and/or chemotherapy (CT), that are tailored to the clinical case according to age, extent of post-surgery residual disease, metastatic status, and molecular and cellular markers[4]. Radiation therapy consists of risk-adapted craniospinal irradiation (CSI) followed by a boost to the whole posterior cranial fossa (WPCF) or to the primary tumor bed (PCF tb-boost) [5]. MB is divided into low, standard and high risk, according to clinical, histological and molecular features [3, 6]. The last European trial SIOP PNET5 MB (already closed for recruitment, although the observation is still ongoing) included reduction of craniospinal irradiation intensity from 23.40 to 18 Gy for low-risk patients and randomization to RT alone or RT concomitant with Carboplatin in standard-risk patients with a classic CSI dose of 23.40 G; in both risk groups a volumetric boost reduction in PCF, from WPCF boost to tumor bed boost, with RT dose up to 54 Gy was provided [6]. These improvements aim to spare the organs at risk minimizing doses to hippocampi, temporal lobes, hypothalamic-pituitary axis, and cochleae, increasing long-term survivors’ clinical outcomes and quality of life.

Radiotherapy planning based on the fusion of pre- and post-surgery brain MRIs and the use of intensity-modulated radiation therapy (IMRT) techniques or proton beam therapy are mandatory in the Protocol [6].

High risk MB treatment consists of high dose CSI up to 39 Gy and often high dose chemotherapy with a stem cell rescue [7]. The MB survivors are at high risk to develop long term late effects, such as neurocognitive and psychosocial impairments, neuroendocrine damage, and alopecia [6, 8].

Permanent alopecia in childhood cancer survivors is a late effect that negatively impacts vitality and social function [9].

The risk of permanent hair loss after radiotherapy has been studied more extensively in adults [9] and increases with radiation therapy and high dose chemotherapy [10, 11]. Due to the large volume involved, the craniospinal irradiation seems to be associated with a higher risk of permanent alopecia [10, 12]. High dose chemotherapy after RT increases the risk of radio-necrosis and other toxicities. However, in long-term childhood cancer survivors its role is still unclear [10, 12–15].

In this study, we analyzed a cohort of MB long-term survivors treated with standard, intermediate and high radiotherapy dose intensity regimes, to assess dose-associated factors that can be predictive of permanent hair loss. Our secondary aim was to assess the possible effect of high dose chemotherapy on the development of alopecia in pediatric patients.

## Materials and methods

### Study criteria

We retrospectively analyzed the clinical records of all consecutive survivors of MB who underwent chemotherapy at Gaslini Children’s Hospital in Genoa, Italy, and radiotherapy at IRCCS Policlinico San Martino Hospital in Genoa, from January 1999 to September 2019. Inclusion criteria were histological diagnosis of MB, completed radiotherapy treatment plan and at least 1-year-long-follow-up after the end of treatment. We excluded patients younger than 3 years of age because they do not receive radiotherapy.

All patients (or their parents if minors) gave written informed consent to undergo RT and accepted that their anonymous data could be used in scientific studies. This study was approved by the local Ethics Committee of IRCCS Policlinico San Martino Hospital of Genoa (n°717/2021).

We collected and retrospectively reviewed clinical and instrumental data of all eligible patients including demographic data, age at time of treatments and at the last follow up, endocrinological screening, oncological treatments and the resulting hair-bearing areas of the head toxicities.

### Oncological treatments

All patients received chemotherapy and radiotherapy according to the oncological protocol in use at the start of the treatment.

Patients were treated with three-dimensional conformal radiation therapy (3D CRT) technique (Varian CLINAC 2100, Treatment Planning System (TPS) Eclipse 13.0 and previous) before 2010, Intensity-Modulated Radiation Therapy (IMRT) has been used for the PCF boost from 2010 and volumetric technique by Helical Tomotherapy (HiArtAccuray TPS) has become the standard treatment from 2015. The radiotherapy plan consisted of CSI (Phase 1) and, according to the protocol in use, PCF boost or WPCF boost (Phase 2). In this second phase, patients received a radiation boost only of tumor bed or of the whole PCF or of both in case of residual disease after surgery. For each radiotherapy plan we retrospectively analyzed prescription data: total dose, number of fractions (dose/die, fraction/die, daily dose) and radiotherapy technique (3D-CRT or IMRT). The Planning Target Volume (PTV) -boost in milliliters (mL) was calculated to assess if higher treatment boost volume was associated with a higher incidence of alopecia.

The photon beam energy used for 3D CRT was mainly 6MV (except for oversize patients) and 6MV in helical tomotherapy. Typical brain beam arrangements in patients treated by 3D-CRT included two fields with 90° and 270° gantry conformed with 1 cm Multileaf collimator (MLC), whereas HiArtTomotheraphy, with 0.287 pitch, included a 2.5 cm field and modulation factor ranging from 2–3.

Patients were all treated supine, with immobilization masks on both face and body, and positioning tattoos on the trunk and abdomen-pelvis region.

We classified the patients in 3 groups, according to the class of risk, based on CSI dose: standard risk dose, adapted dose, and high risk dose. Supplementary table 1 shows the radiotherapy dose adopted.

### Alopecia

Alopecia was assessed in the scalp and nape region, based on irradiated volume (Supplementary Fig. 1), according to the Common Terminology Criteria for Adverse Events (CTCAE) v5.0 rating scale [14]: G1 (less than 50% hair loss and/or possibility of camouflage with other hair) and G2 (greater than 50% loss, no possibility of camouflage, use of wigs/other aids necessary). It was determined by physical examination performed by the oncologist (A.V.) and defined as permanent if persistent longer than 1 year [15]. For each patient, we examined the other hair-bearing areas of the head: the eyebrows and the eyelashes.

### Statistical analysis

Data analysis was conducted by using SPSS® software version 26 (IBM Corp. Released 2019. IBM SPSS Statistics for Windows, Version 26.0. Armonk, NY: IBM Corp). Dichotomous and continuous variables were compared with the chi squared test and by using a one way analysis of variance (ANOVA), respectively.

Univariate and multivariate binary logistic regression analysis with a stepwise backward procedure was performed. In the multivariate model were included all the variables with a p-value < 0.10 at the univariate analysis. The occurrence of permanent alopecia of the whole scalp and the one of the nape region were set as dependent variables. The same analysis was conducted considering separately and jointly (sum group) the grade of severity (grade 1 and grade 2) of alopecia in both two groups. In the alopecia whole scalp group, the PCF dosimetry data were not considered.

Odds ratio (OR) were reported with the respective 95% confidence interval (CI).

Statistical significance was set at p < 0.05.

## Results

We analyzed a total of 41 patients with MB with a median follow-up of 116 months (range: 15—250). Twenty-three (56%) were males and 18 (44%) were females. The mean age at diagnosis was 7.09 years (range: 2- 16). The mean age at last follow up was 17 years (range: 6–30). The demographic and clinical features of the patients, including endocrinological results, related to the grade of alopecia, are reported in Table 1.

**Table 1:** Demographic, clinical and dosimetric variables

	Overall n = 41	Grade of alopecia			
		G0 n = 19 (46%)	G1 n = 11 (27%)	G2 n = 11 (27%)	p-value G0 vs. G1/G2
Age at diagnosis (years)	7 (2–16)				0.592
HD-CT (n = 22)					0.029
Thiotepa	14 (64)	3 (14)	3 (14)	8 (36)	
Others	8 (36)	5 (23)	2 (9)	1 (5)	
Timing HD-CT (n = 22)					0.001
Pre-RT	6 (28)	3 (14)	3 (14)	0	
Post-RT	8 (36)	0	0	8 (36)	
Unknown	8 (36)	5 (23)	2 (9)	1 (5)	
RT-Technique CSI					0.336
3D-CRT	29 (71)	12 (63)	9 (82)	8 (73)	
IMRT-Hel	12 (29)	7 (37)	2 (18)	3 (27)	
Total Dose CSI					< 0.0001
39.00 Gy (HD)	6 (14.5)	0	0	6 (54.5)	
36.00 Gy (HD)	6 (14.5)	1 (5)	5 (45.5)	0	
25.20–31.20 Gy (AD)	9 (22)	4 (21)	1 (9)	4 (36.5)	
23.40 Gy (SD)	20 (49)	14 (74)	5 (45.5)	1 (9)	
Daily dose CSI					< 0.0001
1.80 Gy	24 (59)	15 (79)	6 (54.5)	2 (18)	
2.00 Gy	5 (12)	1 (5)	5 (45.5)	0	
2.60 Gy	12 (29)	3 (16)	0	9 (82)	
PCF boost					0.345
Tumor-bed	14 (34)	7 (37)	2 (18)	5 (45.5)	
W-PCF	27 (66)	12 (63)	9 (82)	6 (54.5)	
RT-Technique boost					0.116
3D-CRT	23 (56)	10 (53)	9 (82)	4 (36.5)	
IMRT-Helicoidal	18 (44)	9 (47)	2 (18)	7 (64)	
Total dose tumor bed					0.059
≤ 54.00 Gy	13 (32)	9 (47)	2 (18)	2 (18)	
> 54.00 Gy	28 (68)	10 (53)	9 (82)	9 (82)	
Hypothyroidism					0.277
Primary	12 (29)	5 (27)	2 (18)	5 (45.5)	
Secondary	2 (5)	1 (5)	0	1 (9)	
Mixed	4 (10)	1 (5)	2 (18)	1 (9)	
No	23 (56)	12 (63)	7 (64)	4 (36.5)	
Hypogonadism					0.437
Hypergonadotropic	12 (29)	4 (21)	3 (27)	5 (45.5)	
Hypogonadotropic	1 (3)	1 (5)	0	0	
No	28 (68)	14 (74)	8 (73)	6 (54.5)	
Hypovitaminosis D					0.146
Yes	21 (51)	9 (47)	8 (73)	4 (36.5)	
No	20 (49)	10 (53)	3 (27)	7 (64)	
Deficit GH					0.891
Yes	24 (59)	11 (58)	7 (64)	6 (54.5)	
No	17 (41)	8 (42)	4 (36)	5 (45.5)	
Adrenal insufficiency					0.231
Yes	12 (29)	4 (21)	3 (27)	5 (45.5)	
No	29 (71)	15 (79)	8 (73)	6 (54.5)	
Total number of endocrine abnormalities					0.477
5	1 (3)	0	1 (9)	0	
4	4 (10)	2 (10.5)	0	2 (18)	
3	9 (22)	4 (21)	3 (27)	2 (18)	
2	11 (27)	6 (31.5)	2 (18)	3 (27)	
1	13 (32)	4 (21)	5 (46)	4 (37)	
No	3 (6)	3 (16)	0	0	

Statistical significance was set at p < 0.05

CTCAE scale v5.0 G1 mild alopecia, G2 severe alopecia, G0 no alopecia

Age is expressed in mean and range, all other data are expressed in absolute numbers and percentages

HD-CT and Timing HD-CT are on 22 patients, all other data are on 41 patients

The total patient population (41 or 22) was divided by the degree of toxicity manifested, based on the predisposing risk factor, and the percentages were calculated based on the degree of toxicity

Endocrine abnormalities are at the time of last follow up

*HD* high dose, *AD* adapted dose, *SD* standard dose, *HD-CT* high dose chemotherapy, *RT* radiotherapy, *CSI* craniospinal irradiation, *PCF* posterior cranial fossa, *GH* growth hormone

Whole scalp alopecia was observed in 22 patients (54%), equally divided between G1 and G2 (11 each); among the 11 with G1, 7 presented a G2 alopecia in the nape region too.

Nape region alopecia was observed in 22 patients (54%). 19 patients (46%) had no signs of permanent hair loss (G0) neither in the whole scalp nor in the nape region.

There were no cases of permanent hair loss of eyebrows or eyelashes.

All patients received chemotherapy. Furthermore, patients (n = 22) with high risk MB received two courses of high dose chemotherapy: 14/22 patients (64%) received Thiotepa [8 patients (57%) after the end of radiotherapy and 6 (43%) before the start], 8/22 patients (36%) received other chemotherapies such as Etoposide, Carboplatin, Methotrexate.

Craniospinal irradiation with a median dose of 25.20 Gy and PCF boost with a dose up to 69 Gy were administered to all patients.

Every clinical and dosimetric variable was included in the univariate analysis, in order to assess their association with the risk of permanent hair loss of the whole scalp (Table 2) or of the nape region (Table 3).

**Table 2:** Univariate and multivariate analyses considering the development of the alopecia of the whole scalp (G1 and G2, panel A; G1 only, panel B; G2 only, panel C) as the dependent variable

		Univariate analysis			Multivariate analysis		
		OR	95% CI	P value	OR	95% CI	P value
A	Age at diagnosis	1.04	0.87—1.24	NS			
	Protocol 1	0.39	0.08—1.91	NS			
	Protocol 3	2.75	0.25—30.51	NS			
	Protocol 4	0.92	0.05—16.49	NS			
	High dose CT	3.00	0.83 -0.72	0.091	3.90	0.80 – 18.88	NS
	CSI standard dose	1 (Ref.)					
	CSI adapted dose	1.87	0.36—9.48	NS			
	CSI high dose	25.66	2.68—245.84	0.005	30.44	2.85–325.05	0.005
	CSI dose/fr daily	0.97	0.95—0.99	0.039	1.02	0.98 -1.07	NS
	CSI daily dose	1.01	0.99—1.03	NS			
	CSI RT technique	2.83	0.69—11.59	NS			
	Total D tumor bed	1.00	1.00—1.01	0.027	1.00	0.99–1.00	NS
B	Age at diagnosis	0.98	0.79—1.22	NS			
	Protocol 1	1.57	0.25—10.09	NS			
	Protocol 3	7.33	0.48—111.18	NS			
	Protocol 4	3.66	0.17—77.55	NS			
	High dose CT	1.25	0.28—5.52	NS			
	CSI standard dose	1 (Ref.)					
	CSI adapted dose	0.56	0.05—6.03	NS			
	CSI high dose	14.00	1.29 – 150.88	0.030	14.00	1.29–150.88	0.030
	CSI RT technique	0.33	0.06 – 1.96	NS			
	Total D tumor bed	1.00	1.00–1.01	0.053	1.00	0.99–1.00	NS
C	Age at diagnosis	1.12	0.89—1.41	NS			
	Protocol 2	1.22	0.07 – 22.41	NS			
	High dose CT	13.50	1.42 -128.26	0.023	13.83	0.89–213.44	NS
	CSI standard dose	1 (Ref.)					
	CSI adapted dose	8.40	0.70–100.59	0.093	11.69	0.74–183.68	NS
	CSI high dose	84.00	4.47 -1576.52	0.003	79.55	3.06 – 2062.72	0.008
	CSI dose/fr daily	0.96	0.93–0.99	0.018	1.79	0.00—0.01	NS
	CSI daily dose	1.03	1.01–1.06	0.005	1.02	0.97 – 1.07	NS
	CSI RT technique	0.37	0.06–2.24	NS			
	Total D tumor bed	1.00	1.00–1.01	0.057	0.99	0.99—1.00	NS

*OR* odds ratio, *CI* confidence interval, *NS* not significant, *CT* chemotherapy, *CSI* craniospinal irradiation, *fr* fraction, *D* dose, *RT* radiotherapy

Protocol 1 metastatic Medullobastoma and/or unfavourable histology and PNET, Protocol 2 SIOP PNET 4, Protocol 3 AIEOP infants, Protocol 4 AIEOP Medulloblastoma standard risk > 3 years and Central Nervous System tumor’99, CSI RT technique 3D-CRT and IMRT helicoidal. CSI standard dose 23.40 Gy, CSI adapted dose 25.20 Gy or 30.60 Gy or 31.20 Gy, CSI high dose 36 Gy or 39 Gy

Statistical significance was set at p < 0.05

**Table 3:** Univariate and multivariate analyses considering the development of permanent alopecia of the posterior cranial fossa (G1 and G2, panel A; G1 only, panel B; G2 only, panel C) as the dependent variable

		Univariate analysis			Multivariate analysis		
		OR	95% CI	P value	OR	95% CI	P value
A	Age at diagnosis	1.02	0.86—1.22	NS			
	Protocol 1	0.33	0.06—1.61	NS			
	Protocol 3	2.30	0.21—25.66	NS			
	Protocol 4	0.77	0.04—13.87	NS			
	High dose CT	2.40	0.68—8.47	NS			
	CSI standard dose	1 (Ref.)					
	CSI adapted dose	2.91	0.57—14.82	NS			
	CSI high dose	25.66	2.68—245.84	0.005	25.66	2.68—245.84	0.005
	CSI dose/fr daily	0.97	0.94—1.04	NS			
	CSI daily dose	1.02	1.00—1.04	0.050	1.01	0.98—1.04	NS
	CSI RT technique	0.50	0.13—1.97	NS			
	Total D tumor bed	1.00	1.00—1.01	0.040	0.99	0.99—1.00	NS
	WPCF boost	2.00	0.27—14.69	NS			
	Tumor bed boost	1.11	0.28—4.42	NS			
	PCF RT technique	0.76	0.22—2.65	NS			
	PCF dose	0.99	0.99—1.00	NS			
	PCF dose/fr daily	0.97	0.95—1.00	0.063	1.00	0.00—5.00	NS
	PCF daily dose	1.01	1.00 -1.03	0.053	1.00	0.99—1.00	NS
	PCF_PTV Volume	1.00	0.99—1.01	NS			
B	Age at diagnosis	1.08	0.83 -1.40	NS			
	Protocol 1	0.47	0.04—5.57	NS			
	Protocol 2	3.33	0.15—70.90	NS			
	High dose CT	0.97	0.12—6.82	NS			
	CSI standard dose	1 (Ref.)					
	CSI adapted dose	7.00	0.49—98.60	NS			
	CSI high dose	28.00	1.20—648.80	0.038	28.00	1.20—648.80	0.038
	CSI dose/fr daily	0.98	0.95—1.061	NS			
	CSI daily dose	1.00	0.97—1.03	NS			
	CSI RT technique	1.14	0.15—8.59	NS			
	Total D tumor bed	1.00	0.99—1.00	NS			
	WPCF boost	1.16	0.15—9.00	NS			
	PCF RT technique	0.74	0.10—5.49	NS			
	PCF dose	0.99	0.99—1.00	0.067	0.99	0.99—1.00	NS
	PCF dose/fr daily	0.98	0.95—1.02	NS			
	PCF daily dose	1.00	0.98—1.02	NS			
	PCF_PTV Volume	1.00	0.98—1.02	NS			
C	Age at diagnosis	1.00	0.82—1.22	NS			
	Protocol 1	0.28	0.04—1.72	NS			
	Protocol 3	2.00	0.15—25.75	NS			
	Protocol 4	1.00	0.05—18	NS			
	High dose CT	3.30	0.82—13.18	0.091	4.84	0.80—29.19	NS
	CSI standard dose	1 (Ref.)					
	CSI adapted dose	2.10	0.34—12.85	NS			
	CSI high dose	25.20	2.51—252.49	0.006	33.20	2.73—402.53	0.006
	CSI dose/fr daily	0.96	0.94—0.99	0.016	1.02	0.95—1.08	NS
	CSI daily dose	1.02	1.00—1.04	0.029	1.18	0.00—2.50	NS
	CSI RT technique	0.36	0.07—1.74	NS			
	Total D tumor bed	1.00	1.00—1.01	0.031	1.00	0.99–1.00	NS
	Tumor bed boost	1.08	0.24—4.95	NS			
	WPCF boost	2.80	0.36—21.72	NS			
	RT technique	0.77	0.20—2.91	NS			
	PCF dose	0.99	0.99—1.00	NS			
	PCF dose/fr daily	0.96	0.93—0.99	0.044	1.00	0.00—6.02	NS
	PCF daily dose	1.01	1.00—1.02	0.035	1.00	0.01—2.05	NS
	PCF_PTV Volume	1.00	0.98—1.01	NS			

*OR* odds ratio, *CI* confidence interval, *NS* not significant, *CT* chemotherapy, *CSI* craniospinal irradiation, *fr* fraction, *D* dose, *RT* radiotherapy, *PCF* posterior cranial fossa, *PTV* planning target volume

Protocol 1 metastatic Medullobastoma and/or unfavourable histology and PNET, Protocol 2 SIOP PNET 4, Protocol 3 AIEOP infants, Protocol 4 AIEOP Medulloblastoma standard risk > 3 years and Central Nervous System tumor’99, CSI and PCF RT technique 3D-CRT and IMRT helicoidal. CSI standard dose 23.40 Gy, CSI adapted dose 25.20 Gy or 30.60 Gy or 31.20 Gy, CSI high dose 36 Gy or 39 Gy

Statistical significance was set at p < 0.05

## Alopecia of the whole scalp

Considering the alopecia sum group, at the univariate analysis, high dose chemotherapy (OR 3.000, 95% CI 0.839–10.727), high dose of CSI (OR 25.667, 95% CI 2.680–245.842), CSI daily dose per fraction (OR 0.976, 95% CI 0.955–0.999), and total dose at the tumor bed (OR 1.002, 95% CI 1.000–1.003) resulted eligible for multivariate analysis (*p-value* < 0.1 for all the variables). The multivariate model confirmed only the high dose of CSI as an independent predictor of the development of the whole skin alopecia (*p-value* 0.005).

Considering the alopecia G1 group, the univariate analysis identified high dose of CSI (OR 14.000, 95% CI 1.299–150.889) and total dose at the tumor bed (OR 1.002, 95% CI 1.000–1.003) as possible predictors (for both, *p-value* < 0.1). Again, at the multivariate analysis only high dose of CSI resulted an independent prognostic factor (*p-value* 0.030). Similarly, the univariate analysis detected high dose chemotherapy (OR 13.500, 95% CI 1.421–128.258), high dose of CSI (OR 84.000, 95% CI 4.476–1576.519), CSI daily dose per fraction (OR 0.961, 95% CI 0.929–0.993), CSI daily dose (OR 1.035, 95% CI 1.011–1.060), and total dose at the tumor bed (OR 1.002, 95% CI 1.000–1.004) as possible predictors of the development of G2 alopecia (*p-value* < 0.1 for all the variables), but only high dose of CSI reached significance at the multivariate model (*p-value* 0.008).

## Alopecia of the nape region

The univariate analysis showed high dose of CSI (OR 25.667, 95% CI 2.680–245.842), CSI daily dose per fraction (OR 0.972, 95% CI 0.949–0.996), CSI daily dose (OR 1.020, 95% CI 1.000–1.040), PCF daily dose per fraction (OR 0.973, 95% CI 0.944–1.001) and total dose at the tumor bed (OR 1.002, 95% CI 1.000–1.003) as possible predictors of the development of PCF alopecia (*p-value* < 0.1 for all the variables). At the multivariate analysis, only the high dose of CSI resulted an independent prognosticator (*p-value* 0.005).

In patients with G1 PCF alopecia, high dose of CSI (OR 28.000, 95% CI 1.208–648.809) and PCF dose (OR 0.997, 95% CI 0.993–1.000) resulted possible predictors at the univariate analysis (for both, *p-value* < 0.1). The multivariate model confirmed only high dose of CSI as an independent prognostic factor (*p-value* 0.038). Analogously, the univariate analysis identified high dose chemotherapy (OR 3.300, 95% CI 0.826–13.181), high dose of CSI (OR 25.200, 95% CI 2.515–252.497), CSI daily dose per fraction (OR 0.969, 95% CI 0.944–0.994), CSI daily dose (OR 1.023, 95% CI 1.002–1.044), and total dose at the tumor bed (OR 1.002, 95% CI 1.000–1.003) as possible predictors of the development of G2 alopecia of the PCF (*p-value* < 0.1 for all the variables), but the only independent prognosticator at the multivariate analysis was high dose of CSI (*p-value* 0.006).

## Discussion

In our study, we have analyzed a cohort of MB survivors treated with radiotherapy to define the prevalence of permanent alopecia and the risk factors for its onset.

Permanent hair loss was observed in 54% patients. Among several clinical parameters assessed, only the high dose CSI (36 to 39 Gy) emerged as significant predictor of the development of alopecia G1 and G2, independently from the boost dose delivered to the tumor bed above CSI dose, the daily dose administered, the radiotherapy technique applied (3D-CRT vs IMRT-helicoidally), the boost volume and the use of high doses chemotherapy pre- or post-radiotherapy. Exceeding these doses of radiotherapy, the other risk factors lose their significance.

Previous reports showed a dose–response relationship describing the possibility of alopecia onset after radiation therapy [16]. In 2004, Lawanda et al. retrospectively examined 26 brain tumor patients and concluded that a cranial field radiotherapy dose of 43 Gy was associated with onset of permanent alopecia in 50% of patients [17].

Min et al. in 2014 reported on 12 pediatric patients with MB the occurrence of permanent alopecia after craniospinal proton therapy and chemotherapy. Based on their results, the mean dose of ~ 21 Gy (RBE-Relative Biological Effectiveness) could be considered the threshold for permanent alopecia in patients treated with high dose chemotherapy, while the threshold dose for alopecia could be 30 Gy (RBE) with conventional chemotherapy [10].

Scoccianti et al. in 2020, in a large prospective observational study including 101 brain tumor patients (adults and children), identified V40Gy < 5.4 cc as the threshold dose for permanent hair loss. They also hypothesized that Volumetric Modulated Arc Therapy (VMAT), a technique allowing highly conformal dose, is the most appropriate for reducing the dose to the scalp [18].

**Fig. 1:**
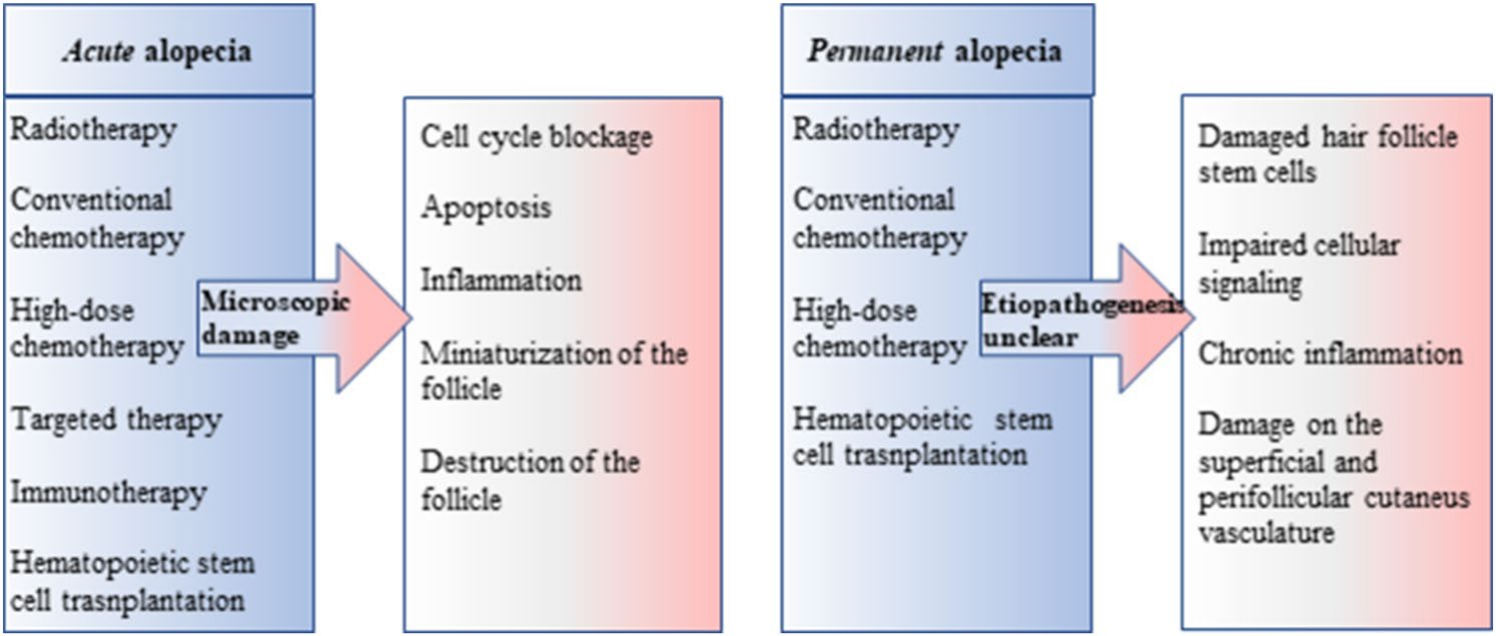
Etiopathogenesis of hair loss in children underwent oncological treatments

Based on our results, high dose CSI of ≥ 36 Gy is the only risk factor for development of G1 and G2 permanent alopecia in the whole-scalp as well as in the PCF region, independent of other clinical and therapeutic variables. The higher dose of the PCF boost was not found to be a significant risk factor for developing alopecia, even in the subgroup of patients with alopecia limited to the posterior cranial fossa. These results confirm that the homogeneous dose distribution for a large scalp volume increases the sensitivity of the hair follicles to the dose and thus decreases the tolerated dose level [10, 17, 19]. Oncological treatment-induced alopecia has multifactorial etiopathogenesis, as shown in Fig. 1 the causes of permanent alopecia are unclear [20].

Our results support the assumption that the high dose chemotherapy does not influence the occurrence of permanent alopecia in patients treated with high dose radiotherapy, the presence of eyelashes and eyebrows in our cohort could be a clinical indicator of this result [20]; it is however conceivable that in patients treated by low or standard dose CSI, the high dose chemotherapy could play a role in the permanent hair tinning onset.

**Fig. 2:**
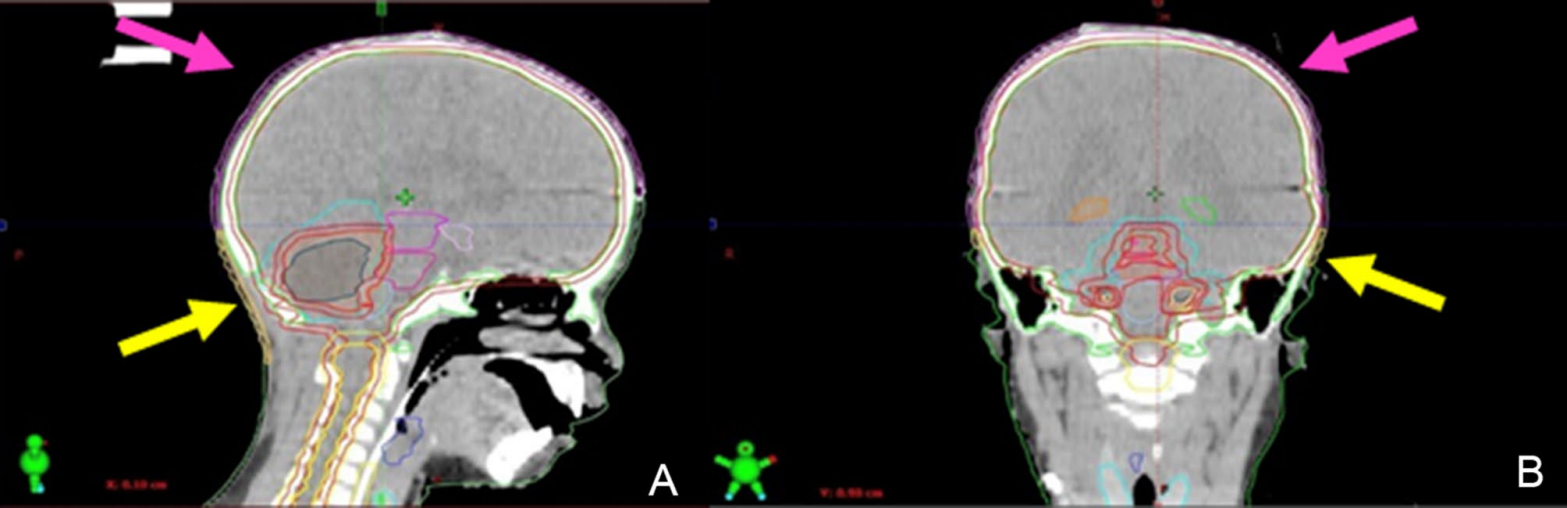
Original CT treatment plan scalp contouring. A Saggital and (B) Coronal view, whole-scalp ROI is pink colored and nape ROI is yellow colored. The thickness of skin contouring ranged from 3 to 5 mm based on the thickness of the skin. To determine the deep contour of the scalp we used the bone window, for the outer limit the soft tissue window

Therefore, in order to confirm the data identified by Min et al. and from Scoccianti et al. as relevant factors in development of permanent alopecia, it is necessary to carry out a further study in a larger cohort of patients with a CSI dose lower than 36 Gy, in order to statistically assess whether other parameters may also influence the onset of permanent alopecia. In these patients, a specific scalp contouring as shown in Fig. 2 could allow dose scalp sparing without uncovering the tumor target.

The strengths of our study are represented by the fact that our cohort consisted of a homogeneous sample of MB survivors (especially by histology, therapy, and age group), treated with CSI and PCF boost with a median very long-term follow-up (9.6 years), subjected to statistical analysis.

On the other hand, this work presents two main limitations: retrospective and monocentric design and the limited number of subjects, due to the fact that MB is a rare disease.

However, despite these limitations, we identified a statistically significant CSI dose as the single permanent alopecia determinant.

## Conclusion

Our study identifies high doses radiotherapy as an independent risk factor for the development of permanent alopecia G1 and G2. Parents and patients undergoing MB high risk treatment, with high doses radiotherapy, should be always informed of this unavoidable risk.

Future studies are necessary to identify the main risk factors for the development of permanent alopecia G1-G2 for patients undergoing standard dose and low dose CSI. For this purpose, whole scalp and PCF contouring is a useful tool which allows conforming the dose to the scalp without discovering the target.

## Supplementary Information

Below is the link to the electronic supplementary material.

Supplementary file1 (DOCX 1546 kb)

## Data Availability

The datasets generated during and/or analyzed during the current study are available from the corresponding author on reasonable request.

## Abbreviations

CNS: Central Nervous System
CP: Cyclophosphamide
CSI: Craniospinal Irradiation
CT: Chemotherapy
CTCAE: Common Terminology Criteria for Adverse Events
CTV: Clinical Target Volume
D: Dose
fr: fraction
G: Grade
Gy: Gray
HD-CT: High dose chemotherapy
IMRT: Intensity Modulated Radiation Therapy
MB: Medulloblastoma
MLC: Multileaf collimator
MV: Megavolt
PCF: Posterior Cranial Fossa
PTV: Planning Target Volume
QoL: Quality of Life
RT: Radiotherapy
TPS: Treatment Planning System
VMAT: Volumetric Modulated Arc Therapy
V40: The percentage volume that received at least 40 Gy
WPCF: Whole Posterior Cranial Fossa
3D-CRT: Three-dimensional conformal radiation therapy

## References

[1] Packer RJ, Gajjar A, Vezina G (2006). Phase III Study of Craniospinal Radiation Therapy Followed by Adjuvant Chemotherapy for Newly Diagnosed Average-Risk Medulloblastoma. JCO.

[2] Louis DN, Perry A, Reifenberger G (2016). The 2016 World Health Organization Classification of Tumors of the Central Nervous System: a summary. Acta Neuropathol.

[3] Louis DN, Perry A, Wesseling P (2021). The 2021 WHO Classification of Tumors of the Central Nervous System: a summary. Neuro Oncol.

[4] Bergthold G, Kababri ME, Varlet P (2014). High-dose busulfan-thiotepa with autologous stem cell transplantation followed by posterior fossa irradiation in young children with classical or incompletely resected medulloblastoma: High Risk Localized Medulloblastoma Outcome. Pediatr Blood Cancer.

[5] Lannering B, Rutkowski S, Doz F (2012). Hyperfractionated Versus Conventional Radiotherapy Followed by Chemotherapy in Standard-Risk Medulloblastoma: Results From the Randomized Multicenter HIT-SIOP PNET 4 Trial. JCO.

[6] Mynarek M, Milde T, Padovani L (2021). SIOP PNET5 MB Trial: History and Concept of a Molecularly Stratified Clinical Trial of Risk-Adapted Therapies for Standard-Risk Medulloblastoma. Cancers.

[7] Bailey S, André N, Gandola L (2022). Clinical Trials in High-Risk Medulloblastoma: Evolution of the SIOP-Europe HR-MB Trial. Cancers.

[8] Rogers S, Donachie P, Sugden E (2011). Comparison of permanent hair loss in children with standard risk PNETS of the posterior fossa following radiotherapy alone or chemotherapy and radiotherapy after surgical resection: Hair Loss after Chemo-Radiotherapy for PNET. Pediatr Blood Cancer.

[9] Kinahan KE, Sharp LK, Seidel K (2012). Scarring, Disfigurement, and Quality of Life in Long-Term Survivors of Childhood Cancer: A Report From the Childhood Cancer Survivor Study. JCO.

[10] Min CH, Paganetti H, Winey BA (2014). Evaluation of permanent alopecia in pediatric medulloblastoma patients treated with proton radiation. Radiat Oncol.

[11] de Jonge M, Mathôt R, Dalesio O (2002). Relationship between irreversible alopecia and exposure to cyclophosphamide, thiotepa and carboplatin (CTC) in high-dose chemotherapy. Bone Marrow Transplant.

[12] Noël G, Antoni D (2022). Organs at risk radiation dose constraints. Cancer/Radiothérapie.

[13] Lafay-Cousin L, Dufour C (2022). High-Dose Chemotherapy in Children with Newly Diagnosed Medulloblastoma. Cancers.

[14] (2017) Common Terminology Criteria for Adverse Events (CTCAE). 155

[15] Lambrecht M, Eekers DBP, Alapetite C (2018). Radiation dose constraints for organs at risk in neuro-oncology; the European Particle Therapy Network consensus. Radiother Oncol.

[16] De Puysseleyr A, Van De Velde J, Speleers B (2014). Hair-sparing whole brain radiotherapy with volumetric arc therapy in patients treated for brain metastases: dosimetric and clinical results of a phase II trial. Radiat Oncol.

[17] Lawenda BD, Gagne HM, Gierga DP, et al (2004) Permanent alopecia after cranial irradiation: Dose–response relationship. International Journal of Radiation Oncology*Biology*Physics 60:879–887. 10.1016/j.ijrobp.2004.04.031 10.1016/j.ijrobp.2004.04.031 15465206

[18] Scoccianti S, Simontacchi G, Greto D (2020). Dosimetric Predictors of Acute and Chronic Alopecia in Primary Brain Cancer Patients Treated With Volumetric Modulated Arc Therapy. Front Oncol.

[19] Eekers DB, in ’t Ven L, Roelofs E,  (2018). The EPTN consensus-based atlas for CT- and MR-based contouring in neuro-oncology. Radiother Oncol.

[20] Kessler S, Marzooq A, Sood A (2022). Alopecia in children undergoing chemotherapy, radiation, and hematopoietic stem cell transplantation: Scoping review and approach to management. Pediatr Dermatol.

