# Supplementary material for: High dose craniospinal irradiation as independent risk factor of permanent alopecia in childhood medulloblastoma survivors: cohort study and literature review
Source: J Neurooncol. 2022 Nov 12;160(3):659–68. doi: 10.1007/s11060-022-04186-2 (PMC9758075; doi:10.1007/s11060-022-04186-2)
Supplement: Supplementary file 1 — Supplementary file1 (DOCX 1546 kb) [file 11060_2022_4186_MOESM1_ESM.docx]

**SUPPLEMENTARY MATERIAL**

Table 1. Craniospinal irradiation dose prescribed.

|  | CSI dose (Gy) | Number of daily fractions | Dose per fraction (Gy) | Daily dose (Gy) |
| --- | --- | --- | --- | --- |
| Standard dose | 23.40 | 1 | 1.80 | 1.80 |
| Adapted dose | 25.20 | 1 | 1.80 | 1.80 |
|  | 30.60 | 1 | 1.80 | 1.80 |
|  | 31.20 | 2 | 1.30 | 2.60 |
| High Dose | 36.00 | 2 | 1.00 | 2.00 |
|  | 39.00 | 2 | 1.30 | 2.60 |

CSI craniospinal irradiation.

Figure 1. Craniospinal irradiation treatment plan.


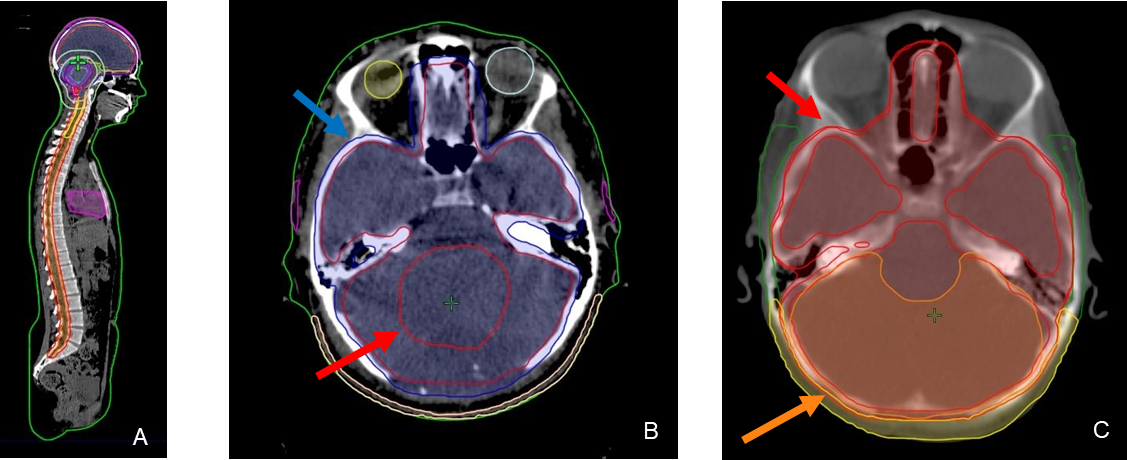


1. Original CSI plan
2. Original whole-cranial (blue) and tumor bed boost in the PCF (red), CTV radiotherapy plan;
3. Original whole-cranial (red) and W-PCF boost (orange), CTV radiotherapy plan.
